# Structural Biology in the AlphaFold Era: How Far Is Artificial Intelligence from Deciphering the Protein Folding Code?

**DOI:** 10.3390/biom15050674

**Published:** 2025-05-06

**Authors:** Nicole Balasco, Luciana Esposito, Luigi Vitagliano

**Affiliations:** 1 Institute of Molecular Biology and Pathology, National Research Council (CNR), c/o Department Chemistry, Sapienza University of Rome, 00185 Rome, Italy; nicole.balasco@cnr.it; 2 Institute of Biostructure and Bioimaging, Department of Biomedical Sciences, National Research Council (CNR), 80131 Naples, Italy; luciana.esposito@cnr.it

**Keywords:** protein structure predictions, sequence–structure paradigm, sequence–stability relationships, CASP

## Abstract

Proteins are biomolecules characterized by uncommon chemical and physicochemical complexities coupled with extreme responsiveness to even minor chemical modifications or environmental variations. Since the shape that proteins assume is fundamental for their function, understanding the chemical and structural bases that drive their three-dimensional structures represents the central problem for an atomic-level interpretation of biology. Not surprisingly, this question has progressively become the Holy Grail of structural biology (the folding problem). From this perspective, we initially describe and discuss the different formulations of the folding problem. In the present manuscript, the folding problem is framed from a historical perspective, effectively highlighting the progress made in the last lustrum. We chronologically summarize the major contributions that traditional methodologies provide in approaching this multifaceted problem. We then describe the recent advent and evolution of predictive approaches based on machine learning techniques that are revolutionizing the field by pointing out the potentialities and limitations of this approach. In the final part of the perspective, we illustrate the contribution that computational approaches will make in current structural biology to overcome the limitations of the reductionist approach of studying individual molecules to afford the atomic-level characterization of entire cellular compartments.

## 1. The Central Role of Proteins in Life and Their Extraordinary Chemical and Structural Complexity

Life, so far detected uniquely on Earth, is an extraordinarily intricate phenomenon based on molecules endowed with uncommon chemical and physicochemical complexities. Interestingly, the atoms constituting these biologically active (macro)molecules present intricate three-dimensional organizations susceptible to even minor chemical modifications or environmental variations. The responsiveness of biomolecules to external stimuli represents a landmark of biology. In this context, proteins represent prototypical examples. Indeed, these macromolecules, which play fundamental roles in all biological processes, are typically formed by thousands of atoms, and even replacing a few of them may have devastating effects on their functionality. Frequently, the activities of these “molecular giants with feet of clay” may be undermined by minimal modifications to the local environments. Fragility is only the apparent weakness of proteins, as it represents a key factor for their ability to play crucial roles in the labyrinth of molecular processes that characterize life. Proteins exploit their structural versatility to react appropriately to external stimuli, establish reversible physiological partnerships, or bind/release substrates and products of reactions. Since the shape that proteins assume is fundamental for their function, understanding the basis that drives their three-dimensional structures represents the central problem for an atomic-level interpretation of biology, in such a way that this question has become the Holy Grail of structural biology [1]. The solution to this problem is complicated by many factors, including the extreme chemical complexity of these molecules, the high variability of the structures they may assume, and the dynamic behavior of their three-dimensional organizations. A simple visual inspection of the repertoire of protein shapes reported in the Protein Data Bank (PDB), an open repository for experimentally resolved structures [2], provides an immediate picture of proteins’ global structural variability. The idea of the intrinsic complexity of this subject can be clarified through the illustration of the so-called Levinthal paradox [3,4]. By making simple assumptions, C. Levinthal showed that even for a relatively small protein of 100 amino acid residues, the astronomical number of possible conformations (at least 3^200^) cannot be explored in the experimental protein folding process even if under physiological conditions each state is sampled at ultrafast speed (e.g., in the order of 10^−15^ s). An obvious consequence of this observation is that the possible conformational states of proteins cannot be exhaustively evaluated using the concepts and methodologies commonly used in structural and computational chemistry.

**Figure 1:**
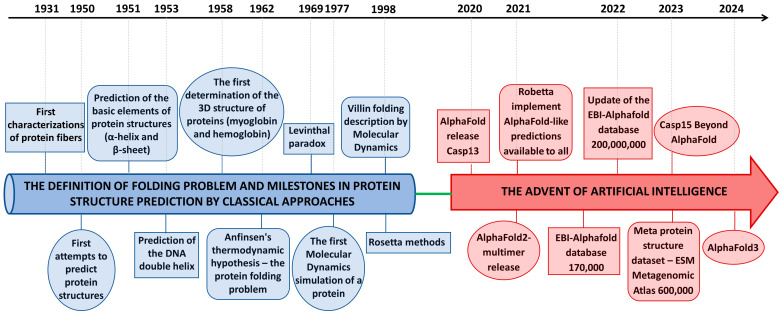
The chronological unfolding of the protein folding story.

However, the possibility, at least theoretically, of tackling the protein folding problem has been suggested by the seminal experiments performed by C. Anfinsen and collaborators. They demonstrated that all the information proteins require to assume well-defined structural organizations resides in their chemistry, i.e., the sequence of amino acid residues constituting their primary structure [5,6,7]. Indeed, they proved that proteins could be refolded in the test tube after removing the chemical species that induced their unfolding, thus showing, at least for the tested cases, that no other cellular component was essential for gaining a native three-dimensional organization. Although several exceptions to this sequence–structure paradigm have been pointed out over the years, it represents a valid working hypothesis widely accepted by the structural biology community. Not surprisingly, the folding problem has become a central structural biology problem [8]. As detailed in the following paragraphs, the definition of this sequence–structure paradigm has also provided conceptual support to the attempts carried out over many decades to predict protein structures using only sequence information. In the present perspective, to better appreciate the importance of recent advancements in the field, the story of the folding problem is illustrated from a historical viewpoint (Figure 1). After briefly describing the so-called folding problem and a rapid excursus of the traditional methods employed to unravel the basis of the sequence–structure relationship, we delineate the impact that machine-based approaches, such as AlphaFold, have on modern structural biology. We highlight these approaches’ strengths and weaknesses by discussing how some structure-oriented biases have paradoxically favored their success. Finally, we show how implementing this approach may significantly contribute to overcoming the limitations of reductionist approaches by allowing the simultaneous study of entire protein families. The fruitful integration of the potential of this approach with innovative structural biology techniques will stimulate future atomic-level studies of the functioning of entire cellular compartments.

## 2. The Protein Folding Code and Its Formulation(s)

The discovery that the amino acid sequence dictates the three-dimensional structure of proteins has generated intense research on the chemical and structural bases of this relationship. Over the years, different views on the mechanism of protein folding have been proposed, based either on pre-defined pathways characterized by well-defined intermediates or on the shape of the energy driving random folding in a reasonable time frame [9]. Deciphering the structural code linking protein sequence and structure is only part of the folding problem, which aims for a comprehensive understanding of the thermodynamic and kinetic factors that drive proteins to assume well-defined three-dimensional organizations. In its most general formulation, elegantly proposed by Dill and coworkers [8], which fully embodies the chemical and structural complexity of proteins, the folding problem deals with three distinct yet related issues. The first is the physical folding code, which focuses on thermodynamic factors, such as the balance of interatomic forces that favor the three-dimensional native structure(s) of a protein. The second issue is unraveling the folding mechanism, i.e., understanding the kinetic aspects of the pathways that allow proteins to fold quickly. The third issue is predicting the protein structure, focusing on computational issues related to determining protein structures using only sequence information. Despite significant advances observed over the years, elucidating these three aspects of the folding problem is largely incomplete.

## 3. From Ab Initio to Empirical Approaches

The first attempts to predict the spatial organization of proteins using (or sometimes misusing) solely chemical information preceded the determination of the first crystallographic structures, as well as the definition of Anfinsen’s sequence–structure paradigm. In this scenario, it may not be surprising that the proposed solutions were far from the correct ones [10], even when they were suggested by the founder of X-ray and protein crystallography [11]. The determination of the first protein structures [12,13] unveiled their non-regular arrangements, which were in contrast with the expected symmetrical organizations suggested by their known propensity to form ordered crystals [14].

Initial attempts to determine the three-dimensional structure of proteins using only sequence information were based on investigating their energy landscape without a priori assuming that they could adopt multiple possible structural states. However, since these approaches strongly rely on key and difficult-to-achieve prerequisites, such as the availability of accurate force fields, the possibility of performing exhaustive conformational samplings, and accurate description of entropic and solvation effects, their success has been extremely limited. On the other hand, integrating limited experimental information with theoretical modeling has been far more effective. The latter approach has been successfully employed, even in the pioneering years of structural biology, by effectively exploiting the limited information collected with X-ray fiber diffraction studies to generate atomic-level accurate models of the basic secondary structure elements of proteins (α-helices and β-sheets) [15,16], fibrous proteins (e.g., collagen) [17,18], and DNA [19]. Over the years, the progressive release of experimental structures and their collection in the PDB [2] represented a valuable treasure of information needed for designing innovative empirical strategies in which experimental data were used as the basis of computational approaches. Although the complexity of the sequence–structure problem hindered general solutions, most successful approaches were based on empirical modeling. This fact is evident from inspecting the results from the Critical Assessment of Structure Prediction (CASP) (https://predictioncenter.org/, accessed on 1 March 2025) [20], which is a biennial protein-folding challenge that employs scrupulous and objective protocols to evaluate proposed structure prediction solutions. In recent decades, the laboratory led by D. Baker has achieved the most significant accomplishments in the field by developing the Rosetta/Robetta package [21]. In this approach, the preferred structures adopted by fragments of the studied protein were initially derived through the mining of the PDB. Then, the fragments were assembled, and the global fold was evaluated based on energetic considerations. This approach proved to be an effective tool that could provide reliable predictions of protein structures in several cases. More importantly, Rosetta has been successfully applied for the de novo design of proteins endowed with many different folding(s) and functions [22]. Compared to the difficulties in predicting the three-dimensional structure of native proteins, the relative ease of designing proteins with desired structures illustrates the tortuous path that the former faced during evolution, being continuously modified and adapted to operate in different biological contexts. Further information on the state of the art of protein structure prediction methods in the pre-AlphaFold era can be found in detailed literature reviews [23,24,25].

## 4. The Advent of Machine Learning Techniques: The AlphaFold Revolution

Over the years, computational studies have exploited the increasingly available experimentally generated three-dimensional protein structures carefully collected and curated in the PDB. They were used to extract the information needed for developing algorithms or as a benchmark for validating the proposed approaches. Applying machine learning to problems in modeling biological systems is over three decades old [26,27]. Considering the commonly used hierarchical description of protein structures, initial studies dealt with predicting protein secondary structures, extensively using deep neural network-based architectures in combination with evolutionary information.

The first successful model using deep learning dates to 1996 when the PHD (profile fed neural network systems from Heidelberg) protein secondary structure prediction method was made available [28]. Since then, the most successful secondary structure predictions have been based on machine learning techniques [29]. The expansion of the available protein structures in the PDB, owing to impressive methodological and technological advances in the appropriate experimental techniques, has enabled the development of ambitious predictive approaches based on machine learning.

DeepMind developed a deep-learning approach based on a specific convolutional neural network known as AlphaFold (AF) by exploiting a vast collection of protein structural data compiled over six decades [30]. Training of AF encompassed combined data from experimental PDB structures and features derived from multiple sequence alignments (MSAs) to predict distances between pairs of atoms. The first version of AF outperformed the other prediction methods by a significant margin during its first CASP event (CASP13, held in 2018) [31,32]. Indeed, AF achieved a summed z-score of 58.2 in predicting the free modeling structures, i.e., those in which no homologous structure was available, whereas the second-best prediction algorithm reached only 36.6. The impressive performance of AF was announced by the most prestigious scientific journal [33,34] and by the general audience media as an achievement that revolutionized structural biology. However, the real impact of AF on the everyday life of structural biologists at this stage was limited by the lack of user-friendly tools that would permit the replication of these astonishing results with other proteins. The revolution was completed in 2021 with the simultaneous release of AlphaFold2 (AF2) [35] and a novel machine learning-based program developed by D. Baker’s group [36]. The latter intentionally replicated the AF protocol to make the approach freely available and implemented it in easy-to-use packages (RoseTTAFold in https://robetta.bakerlab.org/, accessed on 1 March 2025).

In the CASP14 competition [37], AF2 was, again, very successful when it blew away its competitors. This approach correctly predicted most of the target protein structures based only on their sequences. The AF2 best-predicted structures presented a median backbone accuracy of 0.96 Å in terms of root mean square deviation (RMSD) on C^α^ atoms compared with the corresponding experimental ones. Notably, the second-best algorithm reached an accuracy of only 2.8 Å in terms of RMSD. Besides the high accuracy, the AF2 model also provided excellent self-evaluation criteria to estimate the reliability of the generated models, thus enabling the confident use of the predictions. This initiative was followed by the release of the AlphaFold Protein Structure Database [38], jointly developed by Google DeepMind and EMBL-EBI (https://alphafold.ebi.ac.uk/, accessed on 1 March 2025), at present reporting the three-dimensional structures of the individual polypeptide chains of approximately 200 million proteins cataloged in the UniProt Knowledgebase (https://www.uniprot.org/, accessed on 1 March 2025). AF2 no longer employs convolutional neural networks, but a new deep learning architecture, the Transformers, which has been extensively used in the natural language processing field. Moreover, throughout the whole network, this new AF version reinforces the notion of iterative refinement, repeatedly applied to the architecture modules, a feature related to computer vision approaches that remarkably contributed to the accuracy and reduction of training time. Important extensions of AF2 encompass the possibility of generating accurate three-dimensional models of protein homo- and hetero-complexes [39]. Importantly, user-friendly protocols freely available to the community were implemented in Colab [40].

After the appearance of AF, protein structure prediction has experienced tremendous growth with several new methods and applications. At the end of 2022, Meta AI’s team, using a different language model architecture (ESMFold) [41] without employing MSAs, generated a structural database for more than 600 million metagenomic proteins. ESMFold is considerably faster than other predictors. The database known as Evolutionary Scale Modelling (ESM) Metagenomic Atlas (https://esmatlas.com, accessed on 1 March 2025) includes more than 225 million protein structures predicted with high confidence. The release of AF2 has stimulated the development of several variants by different research groups, often designed to solve specific problems. Interestingly, in the CASP15 competition held in 2022, though the most successful groups developed their own ad hoc tools, all of these were based on AF2, at least partly [42,43].

A further step ahead in this ongoing process is the latest release of AlphaFold3 (AF3), in 2024, with its powerful implementations [44]. AF3 allows, for the first time, not only a prediction of polypeptide chains but also complexes of proteins with other proteins, nucleic acids, small molecules, ions, and modified residues, which can all be predicted with remarkable accuracy. It should be mentioned, however, that predictions of RNA structures by artificial intelligence (AI)-based approaches could not repeat the success obtained with protein structures [45].

## 5. AlphaFold and the Folding Problem

It is virtually impossible to summarize the impressive impact of AF and its successors in the life sciences [30,35,46,47,48,49,50,51,52,53]. Several tens of thousands of articles have cited original papers reporting this approach. According to its developers, AF has been used by 2 million researchers in over 190 countries (https://deepmind.google/technologies/alphafold/impact-stories/, accessed on 1 March 2025). Despite many limitations of the approach [54], interrogating the AF and related servers has become an obligatory step in almost every structural biology study. However, a legitimate question is: How far are we from a deep understanding of protein structural properties?

The molecular complexity of proteins and the necessity to fine-tune their function make the definition of a single or a few structural state(s) an important, yet not definitive, step forward. Indeed, complex dynamic behavior, which is highly diverse in the protein realm, is a fundamental aspect about which AF provides little information. Suppose the same concerns are put forward from the perspective of the folding problem, which, in its most complete formulation (see also Section 2), encompasses not only an understanding of the thermodynamics and mechanism of protein folding, but also the prediction of the protein’s three-dimensional structure. The current data suggest that AF and related approaches are close to achieving only the latter goal. While the molecular mechanism underlying protein folding is out of the scope of AF, the accurate prediction of the folded state structure by AF occurs without accurately addressing the related thermodynamic aspects. Indeed, it is well known that AF cannot reproduce the destabilizing effects that single-point mutations induce in the global structure of proteins.

**Figure 2:**
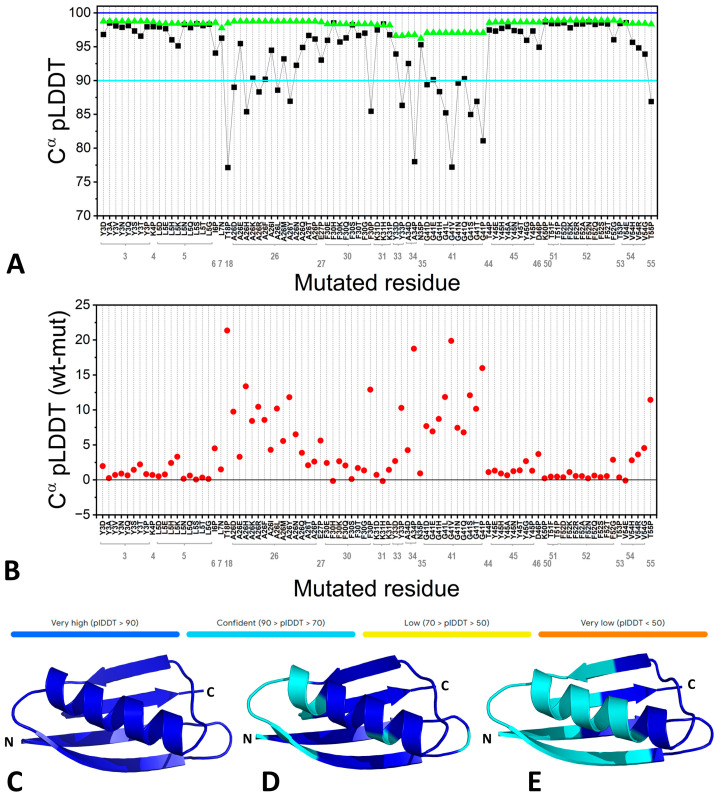
The inability of AF to predict the effects of highly destabilizing mutations in the Gbeta1 protein. Predictions were evaluated using the same parameter employed by AF for self-assessment, namely the per-residue local distance difference test (pLDDT) [56], whose values are reported as horizontal colored bars. (**A**) pLDDT scores for the C^α^ atoms of specific residues of the wild-type protein (wt, green triangles) and single-point mutants (mut, black squares). (**B**) Difference in pLDDT values between wt and single-point mutants. Cartoon representation of the AF3-predicted models of (**C**) wt Gbeta1, (**D**) a mutant protein carrying six destabilizing mutations (Y3A, L5D, A26P, F30G, Y45G, and F52G), and (**E**) a mutant protein carrying eight destabilizing mutations (Y3A, L5D, A26P, F30G, G41P, Y45G, F52G, and V54G). The models are colored according to the AF pLDDT metric.

To further illustrate how far AF stretches in replicating these effects, we consider the protein Gbeta1, a model system for studying protein structure–stability relationships. Previous studies elaborated on the effects of single-point mutations on protein stability and did so for every amino acid residue in the protein [55]. Highly destabilizing mutations preventing the expression and characterization of the protein were identified. According to our predictions, AF provided native-like structures for models simultaneously incorporating up to eight mutations (Figure 2C–E and Table 1), where each could completely destabilize the protein structure in the experiments.

**Figure 3:**
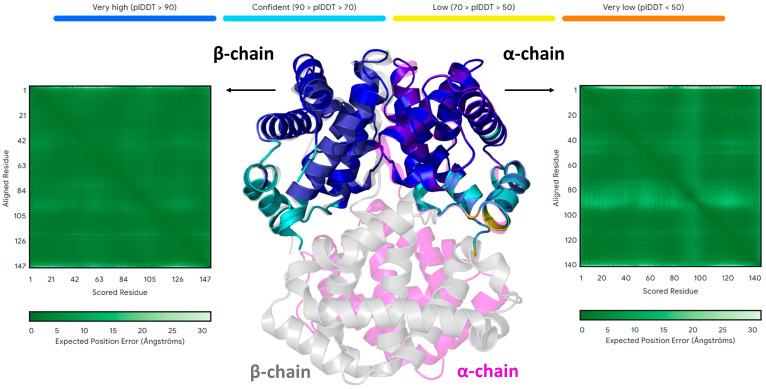
The peculiar ability of AF to predict the structure of individual protein chains without key structural elements, such as prosthetic groups and the oligomeric environment: the case of human hemoglobin (HbA). The AF3-predicted models of the individual HbA chains (central panel), colored according to the AF pLDDT values (upper bar), are superimposed on a couple of the α (magenta) and β (gray) chains of the HbA tetramer from the high-resolution crystallographic structure (PDB entry 2dn2). The associated predicted aligned error (PAE) matrices for the AF3 models are also shown (left and right panels).

The bias toward folded states of AF is well illustrated by the case of human hemoglobin, whose well-known tetrameric structure strongly relies on the heteromeric association of different α and β chains and the presence of the iron-coordinated heme group. As shown in Figure 3, the structures of the individual chains predicted by AF are identical to those detected in tetrameric native human hemoglobin (HbA). Although these results are generally known to the community of AF users, it is somehow overlooked that the inability of AF to catch the real thermodynamics of the folding process has contributed to its success. Indeed, many, if not most, of the protein structures in the AlphaFold DataBase, generated without taking into account the oligomeric state of proteins or the presence of a prosthetic group (such as the heme) or other stabilizing agents, are thermodynamically unstable despite their similarity to experimental native states.

## 6. Conclusions and Perspectives for Structural Biology and Beyond

In the last seven decades, structural biology has experienced several breakthroughs. In its initial stage, structural studies were limited to naturally highly abundant proteins amenable to crystallization. The possibility of producing recombinant proteins has expanded structural biology studies to proteins of extreme biological relevance, which are barely expressed in vivo. Despite spectacular technical and methodological advancements that have significantly increased the pace of solving protein structures in the last few decades, the experimental determination of protein structures is still a bottleneck, i.e., a slow and laborious process with no guaranteed success. The development of effective predictive approaches has completely changed the scenario. While a constantly growing community of several thousand experimental structural biologists took nearly sixty years to determine ~200,000 structures of proteins, individual research groups were able to release hundreds of millions of structures in just a few years. Although these structures need validation, they present a reasonable degree of confidence and accuracy globally. This has opened avenues for other applications aimed at clarifying the role of pathogenic mutations [57] or for the structural interpretation of large-scale interactomes [58]. It is worth noting that the rapid determination of protein structures, starting from their sequences, facilitated by these approaches, allows the simultaneous elucidation of structure–function relationships in entire families, as we have recently shown for KCTD proteins, for which atomic-level structural features were reported for all 25 members of the family [59,60,61].

Clearly, the ability to predict protein structures from their sequences does not diminish the importance of experimental techniques, especially since the latter can yield results that predictive methods cannot achieve [62,63]. In fact, experimental approaches continue to play a crucial role in defining biological processes at the atomic level. Computational methods, on the other hand, cannot (a) achieve the high accuracy of some crystallographic structures [64], (b) address highly complex systems like those investigated by cryo-electron microscopy [65,66], or (c) provide dynamic properties as NMR does [67].

In general, the availability of these predictive methods is an additional opportunity to eliminate the generally used reductionist approaches (which are focused on the characterization of individual biomolecules) and pursue an atomic-level view of life. In the future, integrating experimental and computational methodologies, including some in their early developmental stages, such as tomography, will likely produce atomic-level visualization of entire cellular compartments and their mutual interactions.

In conclusion, the release of AF represents a further step in pursuing an atomic-level view of life. As also indicated by the Nobel Prize in Chemistry awarded in 2024 (https://www.nobelprize.org/prizes/chemistry/, accessed on 1 March 2025), AF currently represents the most successful application of AI to a scientific problem (see also https://www.forbes.com/sites/robtoews/2021/10/03/alphafold-is-the-most-important-achievement-in-ai-ever/, accessed on 1 March 2025). The impact that this approach is having as a solution to the long-standing problem of determining protein three-dimensional structures from their sequence is revolutionary. However, it should be noted that due to the intrinsic nature of AI, its success is not due to conceptual advancement and has not hitherto provided new intellectual interpretive models for the scientific community. If these considerations are placed in Kuhn’s framework of scientific revolution [68], AF release is a revolution without any paradigm change. Instead of “providing model problems and solutions for a community of practitioners” [68], it is a rather effective tool for solving a fundamental scientific problem. The ability of AF to predict protein structures from their sequences reinforces the old folding paradigm and represents a tool revolution that, however, cannot be considered a mere methodological advancement. Hopefully, the treasure of information generated by this methodology will generate new conceptual paradigms that will help humans understand nature. The irresistible advent of AI in science suggests that other revolutions of this type will soon appear.

## Figures and Tables

**Table 1:** Per-residue local distance difference test (pLDDT) [56] (see Figure 2 for the metric) values for the C^α^ atoms of specific residues in the wild-type Gbeta1 protein, in the variants bearing the single-point mutations, and in the mutants incorporating either six (hexa-mutant) or eight (octa-mutant) of these highly destabilizing mutations.

Residue	pLDDT	Mutation	pLDDT in the Single-Point Mutants	pLDDT in the Hexa-Mutant	pLDDT in the Octa-Mutant
Y3	98.76	Y3A	98.51	90.01	89.11
L5	98.43	L5D	97.93	92.94	90.24
A26	98.75	A26P	96.13	89.03	76.83
F30	98.35	F30G	97.00	90.71	86.31
G41	97.06	G41P	81.09	-	93.96
Y45	98.62	Y45G	95.94	92.23	86.23
F52	98.89	F52G	96.02	93.10	88.60
V54	98.44	V54G	93.90	-	92.43

## Data Availability

Not applicable.
